# Ecology of sand flies (Diptera: Psychodidae, Phlebotominae) in Jajarm County, an area with high risk of cutaneous leishmaniasis, in North Khorasan, Iran

**DOI:** 10.1186/s40850-022-00113-0

**Published:** 2022-03-16

**Authors:** Hasan Jalali, Seyed Hassan Nikookar, Nasibeh Hosseini-Vasoukolaei, Elham Jahanifard, Ahmad Ali Enayati, Farzad Motevalli-Haghi, Jamshid Yazdani-Charati

**Affiliations:** 1 Student Research Committee, Faculty of Health, Mazandaran University of Medical Sciences, Sari, Iran; 2 Department of Medical Entomology and Vector Control, Health Sciences Research Center, Addiction Institute, School of Public Health, Mazandaran University of Medical Sciences, Sari, Iran; 3 Department of Medical Entomology and Vector Control, Health Sciences Research Center, Faculty of Health, Mazandaran University of Medical Sciences, Sari, Iran; 4 Department of Medical Entomology and Vector Control, School of Public Health, Ahvaz Jundishapur University of Medical Sciences, Ahvaz, Iran; 5 Department of Biostatistics, Health Sciences Research Center, Faculty of Health, Mazandaran University of Medical Sciences, Sari, Iran

**Keywords:** Sand fly, Ecological aspects, Cutaneous leishmaniasis, Biodiversity, Synanthropic index, northern Khorasan

## Abstract

The present study was conducted to investigate the ecological aspects of sand flies in southwestern North Khorasan, in which cutaneous leishmaniasis caused by *Leishmania major* has been reported with the highest annual incidence in Iran. Sampling was carried out in four localities including: Khorasha (natural), Ghamiteh (natural), Jorbat (semi urban) and Brick kilns (urban), twice a month using 105 sticky paper traps from indoors and outdoors dwellings during May-December 2017. Specimens were removed from sticky papers, washed in acetone, preserved in 80% ethanol, mounted on microscopic slides by Puri’s medium, and identified using valid morphological keys. Simpson (D), richness (S), Menhinick (D_Mg_), Margalef (D_Mn_), Shannon-Weiner (H*′*), evenness (J’) were calculated for species diversity. The synanthropic index was determined for the first time in the area. Totally 517 specimens were collected, 47% in outdoors and 30.4% in human indoor dwellings and 22.6% in animal. Eight species of sand flies including 5 species of the genus *Phlebotomus* and 3 species of the genus *Sergentomyia* were identified. *Phlebotomus papatasi* and *Sergentomyia sintoni* were the most common and Eudominant species, active in all months, collected in the maximum number and percentage in September and August, respectively, and showed the highest abundance in outdoors. The synanthropic index ranged from 6.25 to 38.9 in the study area. The Shannon-Wiener index was estimated to be up to 1.4 and 1.37 in Khorasha and November, respectively, which showed the highest diversity due to maximal richness and evenness compared to other areas. High abundance of *Ph. papatasi*, as the main vector of cutaneous leishmaniasis, can enhance the potential risk of emerging CL in new areas, the data can be equally important when vector control measures are considered.

## Introduction

Phlebotomine sand flies (Diptera: Psychodidae) are tiny dipteran insects which may have wide variety of hosts for blood feeding. They are vectors of several agents of leishmaniasis [1, 2]. Leishmaniasis occurs in most parts of the world, is found in about 89 countries, and is native to Asia, Africa, the Americas, and the Mediterranean region [3]. About 350 million people live in areas at risk for leishmaniasis, and about 2 million new cases are reported worldwide [3]. About 87% of new cases of cutaneous leishmaniasis were reported from Afghanistan, Algeria, Brazil, Colombia, Syria, Libya, Tunisia, Pakistan, Iraq and Iran. Iran is one of the top ten countries in this regard [4].

There are three main form of the disease: visceral leishmaniasis (VL), cutaneous leishmaniasis (CL), and mucocutaneous leishmaniasis (MCL) [4]. Zoonotic Cutaneous Leishmaniasis (ZCL) and Anthroponotic Cutaneous Leishmaniasis (ACL) are common types of leishmaniasis in Iran [5]. Zoonotic Cutaneous Leishmaniasis has a high prevalence in 17 out of 31 provinces of Iran. The main vector of ZCL in Iran is *Phlebotomus papatasi* and its causative agent is *Leishmania major* [6]. Anthroponotic Cutaneous Leishmaniasis is spread in at least 8 provinces of Iran. *Phlebotomus sergenti* is the main vector of ACL in Iran and its agent is *Leishmania tropica* [6, 7]. Cutaneous leishmaniasis has been reported from all counties of North Khorasan province [8, 9].

Approximately 1000 species of Phlebotomine sand flies have been identified worldwide [10, 11]. The first comprehensive study on Phlebotomine sand flies of Iran was conducted in 1964 by Theodor and Mesghali. They reported 34 species of Phlebotomine sand flies, including 20 species of the genus *Phlebotomus* and 14 species of the genus *Sergentomyia* [12, 13]. This list has been increased to 53 species of Phlebotomine sand flies, including 34 species of the genus *Phlebotomus* and 19 species of the genus *Sergentomyia* due to recent studies [14, 15].

In North Khorasan, the first entomological studies began in 1975 in the city of Esfarayen, where *L. major* was detected as the dominant parasite in *Ph. papatasi* [16]. *Phlebotomus kandelakii* was confirmed to be infected with *Leishmania infantum* by molecular methods in Shirvan [17]. Recently, the richness and diversity of Phlebotomine sand flies have been studied in some rural areas of North Khorasan [18].

Species diversity is one of the most important aspects of insect ecology [19]. Species diversity is studied at three levels: Alpha (α), Beta (β) and Gamma (γ). Alpha is the study of biodiversity in one community, has two components - species richness and evenness, while Beta is the comparing of biodiversity between two or more communities. Gama is the study of all types of diversity in an area [20]. There are plenty of indices to study for biodiversity, but the most common are Simpson and Shannon-Wienner [20]. Biodiversity of Phlebotomine sand flies has been done in some provinces of Iran including Qom [21], Khuzestan [22] and West Azerbayjan Province [23].

In general, the behavioral and environmental patterns of sand flies vary in different climates. Knowledge about the ecological aspects of Phlebotomine sand flies can be mentioned as an additional feature for better finding of dynamism of vector and disease between human and reservoirs [20, 24]. Knowledge on the ecology of sand flies is an approach for a proper vector control program. High incidence of CL in Jajarm County, North Khorasan Province give enough importance for doing this study. The annual incidence rate of CL in Jajarm County was in average 237.8/100000 people [25], which was several times higher than the incidence rate of CL in Iran, which was reported averagely 32 /100000 people over 30 year period study in Iran [26]. The aim of this study was to determine ecological aspects of sand flies (Diptera: Psychodidae, Phlebotominae) in an endemic regions of cutaneous leishmaniasis in southwestern North Khorasan, Iran.

## Materials and methods

### Study area

This study was conducted in Jajarm County which is located in southwest of North Khorasan Province between latitude 36°57′00″N, longitude 56°22′48″E and an altitude of 1000 m. It is the most attractive city with several historical and ancient sites, a population of approximately 39,580 and an area of 35,000 km2 (according Census Report in 2016). It is located on the border of the central desert of Iran and has unique vegetation. Geographically, the county is surrounded from the north to Maneh and Samolghan cities, from the west to Garmeh city, from the south to Semnan Province, from the southeast to Razavi Khorasan Province, from the east to Esfarayen city and from the northeast to the city of Bojnurd. The climate is temperate and dry with a mean annual rainfall of 150 mm, relative humidity of 50%, and annual temperature of 16.5 °C. People are mostly occupied in animal husbandry and agriculture.

### Sand fly collection

**Fig. 1:**
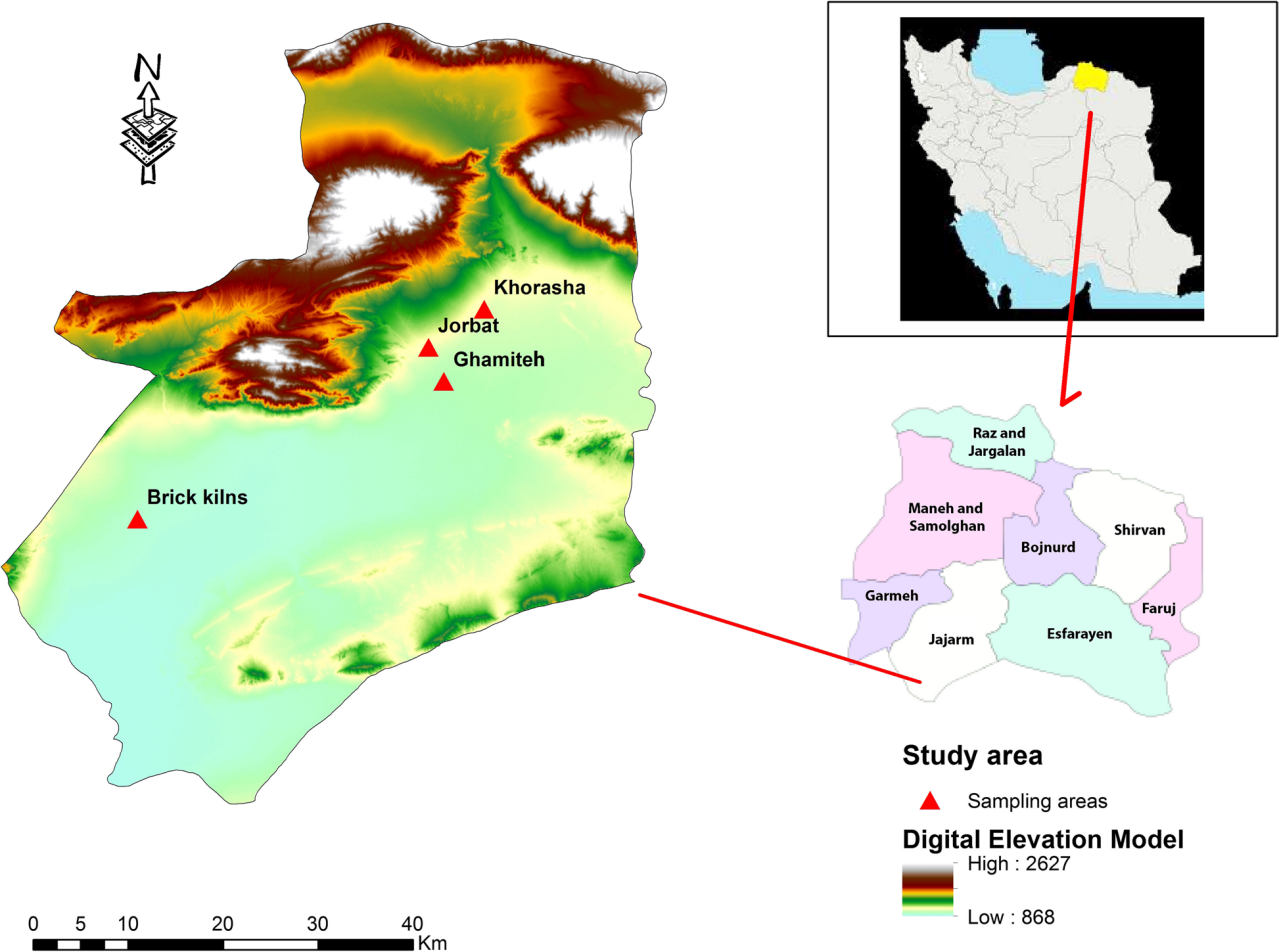
The study area in North Khorasan Province, northeast of Iran, 2017

Sampling was implemented in four localities including: Khorasha (natural), Ghamiteh (natural), Jorbat (semi urban) and Brick kilns (urban) in Jajarm County (Fig. 1), where the cases of ZCL have been confirmed. In each locality, two fixed sites were selected based on their topographic conditions. Specimens were collected twice a month using 105 sticky paper traps from indoors (corners of rooms, storage, bathrooms and toilets, human dwelling and stables) and outdoors (in the cracks of clay walls, yards, around rodents’ nests or animal shelters) from May to December 2017 from fixed places. The traps were installed before sunset and collected the next day before sunrise. The sand flies were separated from sticky papers by needle or brush, washed in acetone, preserved in small vials in 80% ethanol, mounted by Puri’s medium on microscopy slides, and then identified using the morphological-based valid keys [27, 28]. The abbreviations of genera and subgenera of sand flies followed by Galati et al. [29].

### Synanthropic index, dominance structure and sex ratio

Synanthropic index was computed for all species by the following formula:

$$\mathrm{SI}=\frac{\left(2a+b\hbox{-} 2c\right)}{2}$$

Where ‘a’ is the percentage of caught species in the urban area, ‘b’ is the percentage of collected species in the Semi-urban area and ‘c’ is the percentage of collected species in the natural area. The index is ranged between + 100 to − 100; the value of + 100 shows the strong preference of species for anthropogenic settlements and − 100 indicates the lack of preference of species to human dwelling. The average value represents variation degrees of synanthropy [30, 31].

Dominance structure and sex ratio were also calculated for each species in the area. Five categories of dominance structure were considered: Eudominant (ED) (> 10%), Dominant (5-10%), subdominant (SD) (2-5%), recedent (R) (1–2%), and subrecedent (SR) species (< 1%) followed by the following formula:

$$\mathrm{D}=\frac{i}{t}\times 100$$

Where “i” is the total number of specimens of a species and *t* = total samples [32, 33].

### Biodiversity and rarefaction analysis

Indices of species richness, evenness, dominance and diversity were calculated using the Margalef ($DMg=\frac{S-1}{{\mathit{\ln}}_N}$), Menhinick’s ($DMn=\frac{S}{\sqrt{N}}$), Simpson’s dominance (*D = λ =* ${\sum}_{i=1}^S{P_i}^2$), Evenness (J or E or Pielou’s index) ($\mathrm{J}=\frac{H^{\prime }}{H^{\prime}\max }=\frac{H^{\prime }}{\log (S)}\Big)$ and Shannon indices (*H* ′  =  − *Σpi* × *lnpi*) at spatial and temporal scales, where *N* represents the total number of individuals in the sample, *S* represents the number of species in the sample, *P*_*i*_ *=* $\frac{n_i}{N}$; which *P*_*i*_ is the proportion of individuals observed in *i*th species, n_*i*_ is the number of individuals in taxon *i*th and *H*′ is the Shannon-Wiener function [34]. To estimate the sand fly richness and the adequacy of sampling efforts, rarefaction curves have been used, which is shown by the following formula:

$E(Sn)={\sum}_{i=1}^S\left[1-\frac{\left(\genfrac{}{}{0pt}{}{N- Ni}{n}\right)}{\left(\genfrac{}{}{0pt}{}{N}{n}\right)}\right]$, where *N* = total number of individuals in the sample, *S* = total number of species and *N*_*i*_ = number of individuals of species number *i* [35].

### Geoghraphic distribution of sand flies

To map the distribution of the dominant species in this study, the latitude and longitude of each region were recorded with Global Positioning System (GPS), and after identifying the samples, the frequency of this species was added to the table in the Arc map, as the main component of ArcGIS software of geospatial processing program. Then its spatial distribution at different times of the sampling was prepared as a map.

## Result

**Fig. 2:**
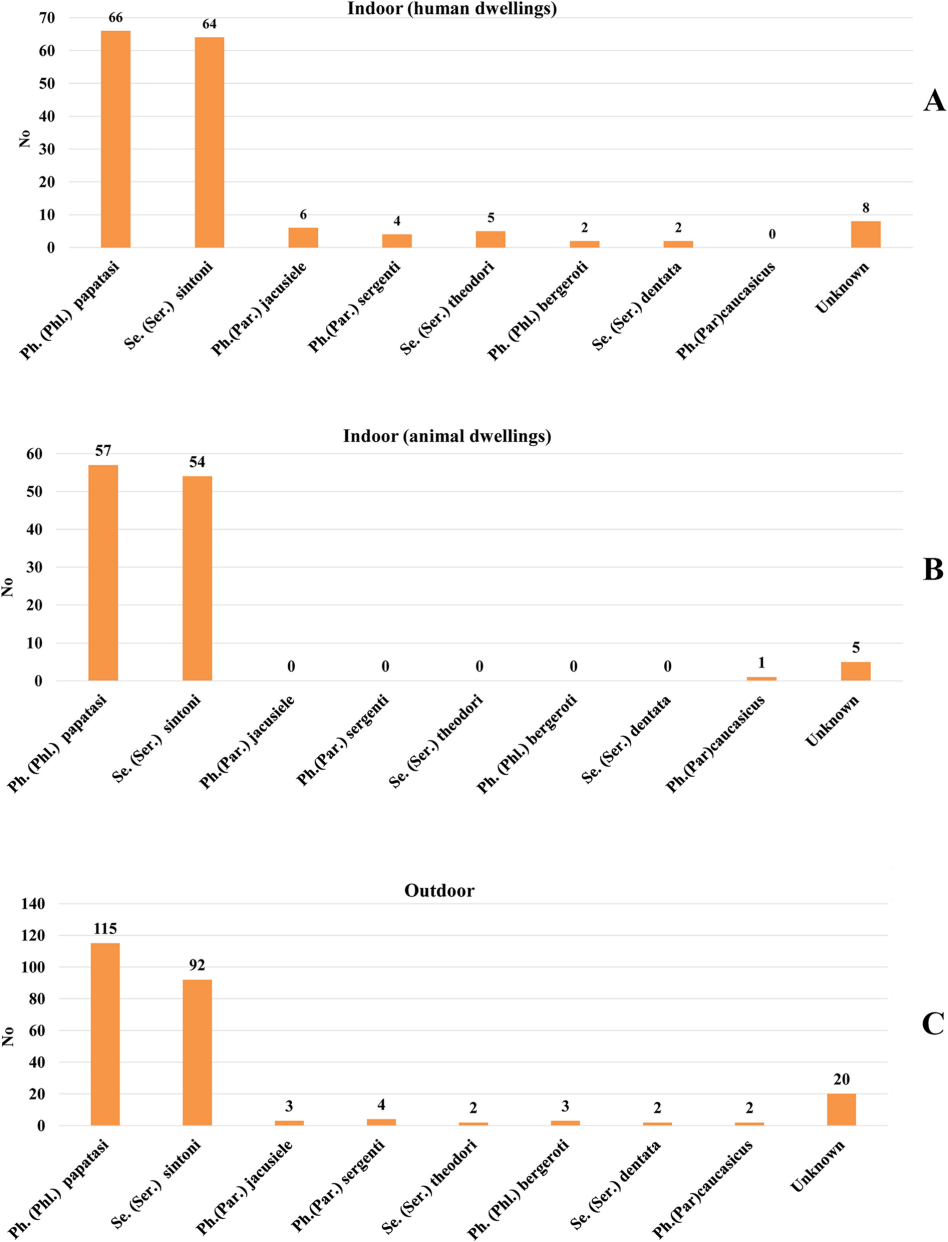
Fauna and number of collected sand flies from (**A**) indoor (human dwellings), (**B**) indoor (animal dwellings), (**C**) outdoor in fixed and random places of Jajarm County, North Khorasan Province, 2017

A total of 517 specimens were collected by sticky paper traps and identified during sampling efforts, of which 5 species belonged to genus *Phlebotomus* from two subgenera: *Phlebotomus* and *Paraphlebotomus*. Three species were classified in the genus and subgenus *Sergentomyia*. Most sand flies were caught from outdoor environments (47%), followed by indoor human (30.4%) and animal (22.6%) dwellings*. Phlebotomus papatasi* and *Se. sintoni* showed the highest abundance in outdoor environments and were Eudominant species up to 46 and 40.6%, respectively. The sex ratio was calculated up to 0.55 for all samples. These values were up to 2.4 and 1.39 for *Phlebotomus* and *Sergentomyia*, respectively. In general, the number of collected females was 64.2% of the total samples (Table 1, Fig. 2).

**Table 1:** Species composition, dominance structure and sex ratio of phlebotomine sand flies in Jajarm County, North Khorasan Province, Iran, May-December 2017

Species	Male		Female		Sex ratio (M:F)	Total	%	Dominance structure
	*N*	%	*N*	%				
*Ph. (Phl.) papatasi*	165	31.9	73	14.1	2.26	238	46	Eudominant
*Se. (Ser.) sintoni*	11	2.13	199	38.5	0.06	210	40.6	Eudominant
*Ph.(Par.) jacusiele*	0	0	9	1.74	0	9	1.74	Recedent
*Ph.(Par.) sergenti*	1	0.19	7	1.35	0.14	8	1.55	Recedent
*Se. (Ser.) theodori*	4	0.77	3	0.58	1.33	7	1.35	Recedent
*Ph. (Phl.) bergeroti*	0	0	5	0.97	0	5	0.97	Subrecedent
*Se. (Ser.) dentata*	0	0	4	0.77	0	4	0.77	Subrecedent
*Ph.(Par.)caucasicus*	0	0	3	0.58	0	3	0.58	Subrecedent
*Unknown*	4	0.77	29	5.61	0.14	33	6.38	Dominant
Total	185	35.8	332	64.2	0.55	517	100	–

Seasonal activity of sand flies began in early May and peaked in August and ended in late December in the study area. The highest abundance of phlebotomine sand flies was observed in August (29.2%), whereas the lowest abundance was in December (1.5%). *Phlebotomus papatasi* and *Se. sintoni* were active in all months and were collected with maximum number and percentage in September and August, respectively. The highest prevalence of sand flies was recorded in summer, followed by autumn and spring. The predominant species, *Ph. papatasi* and *Se*. *sintoni*, were found with the highest number and percentage (n: 147, 61.8% and n: 136, 64.8%) in the warm season. Population fluctuations of other species by season and month are shown in Tables 2 and 3.

**Table 2:** Number and percentage of phlebotomine sand flies in Jajarm County, North Khorasan Province, Iran by collection season, 2017

Species	abundance			Total	%		
	Spring	Summer	Autumn		Spring	Summer	Autumn
*Ph. (Phl.) papatasi*	31	147	60	238	13	61.8	25.2
*Se. (Ser.) sintoni*	33	136	41	210	15.7	64.8	19.5
*Ph.(Par.) jacusiele*	1	5	3	9	11.1	55.6	33.3
*Ph.(Par.) sergenti*	0	3	5	8	0	37.5	62.5
*Se. (Ser.) theodori*	0	6	1	7	0	85.7	14.3
*Ph. (Phl.) bergeroti*	1	2	2	5	20	40	40
*Se. (Ser.) dentata*	1	2	1	4	25	50	25
*Ph.(Par.)caucasicus*	0	3	0	3	0	100	0
*Unknown*	2	18	13	33	6.06	54.5	39.4
Total	69	322	126	517	13.3	62.3	24.4

**Table 3:** Number and percentage of phlebotomine sand flies collected in Jajarm County, North Khorasan Province, Iran by collection month, 2017

Species	May		June		July		August		September		October		November		December	
	*N*	%	*N*	%	*N*	%	*N*	%	*N*	%	*N*	%	*N*	%	*N*	%
*Ph. (Phl.) papatasi*	10	4.2	21	8.8	27	11.3	53	22.3	67	28.2	35	14.7	22	9.2	3	1.26
*Se. (Ser.) sintoni*	5	2.4	28	13	30	14.3	79	37.6	27	12.9	22	10.5	16	7.6	3	1.43
*Ph.(Par.) jacusiele*	1	11	0	0	0	0	4	44.4	1	11.1	1	11.1	2	22	0	0
*Ph.(Par.) sergenti*	0	0	0	0	0	0	1	12.5	2	25	3	37.5	2	25	0	0
*Se. (Ser.) theodori*	0	0	0	0	4	57.1	2	28.6	0	0	0	0	1	14	0	0
*Ph. (Phl.) bergeroti*	0	0	1	20	0	0	0	0	2	40	2	40	0	0	0	0
*Se. (Ser.) dentata*	0	0	1	25	0	0	2	50	0	0	0	0	1	25	0	0
*Ph.(Par.)caucasicus*	0	0	0	0	0	0	0	0	3	100	0	0	0	0	0	0
*Unknown*	1	3	1	3	1	3.03	10	30.3	7	21.2	6	18.2	5	15	2	6.06
Total	17	3.3	52	10	62	12	151	29.2	109	21.1	69	13.3	49	9.5	8	1.55

The synanthropic index for phlebotomine sand flies was in the range of 6.25–38.9 in the study area. The index was up to 26.2 and 7.56 for *Se. sintoni* and *Ph. papatasi*, respectively. The highest and lowest synanthropic indices were estimated for *Ph. jacusiele* (38.9) and *Ph. sergenti* (6.25), respectively. The preference status of other species for compatibility or incompatibility with human settlements is shown in Table 4.

**Table 4:** Synanthropic index of sand fly species collected in natural ecosystem, semi urban and urban habitats in Jajarm County, North Khorasan Province, Iran, May-December 2017

Species	Urban	Semi urban	Natural	Total	SI
*Ph. (Phl.) papatasi*	77	68	93	238	7.56
*Se. (Ser.) sintoni*	92	54	64	210	26.2
*Ph.(Par.) jacusiele*	4	3	2	9	38.9
*Ph.(Par.) sergenti*	2	3	3	8	6.25
*Se. (Ser.) theodori*	3	1	3	7	7.14
*Ph. (Phl.) bergeroti*	3	0	2	5	20
*Se. (Ser.) dentata*	1	2	1	4	25
*Ph.(Par.)caucasicus*	1	1	1	3	16.7
*Unknown*	13	9	11	33	19.7

Biodiversity indices of sand flies at spatial and temporal scales are shown in Tables 5 and 6. The Shannon-Wiener index was estimated to be up to 1.4 and 1.37 in the Khorasha area and November, respectively. Maximum richness (*S*) was revealed in Brick kilns (*S* = 9) and August, September and November (*S* = 7). Menhinick (*D*_*Mg*_) and Margalef (*D*_*Mn*_), as indices of species richness, did not show the highest common numerical values in an area, while they were jointly high in November. The highest values of evenness (*J*’) index were recorded in Khorasha (*J*’ = 0.50) and December (*J*’ = 0.98). While the highest Simpson’s diversity index was observed in Ghamiteh (*D*: 0.41), followed by Brick kilns, Jorbat (*D*: 0.38) and Khorasha (*D*: 0.29), indicating the strong influence of Eudominant species, *Ph. papatasi* on other species in the area.

**Table 5:** Biodiversity indices of sand flies in Jajarm County, North Khorasan Province by Spatial scale, 2017

Species	Ghamiteh	Khorasha	Jorbat	Brick kilns
Richness (*S*)	6	8	8	9
Abundance (*N*)	87	120	141	196
Menhinick (*D*_*Mg*_)	0.64	0.73	0.67	0.64
Margalef (*D*_*Mn*_)	1.12	1.46	1.41	1.51
Shannon- Weiner (*H′*)	1.06	1.4	1.18	1.21
Simpson (*D*)	0.41	0.29	0.38	0.38
Evenness (*J’*)	0.48	0.50	0.41	0.37

**Table 6:** Biodiversity indices of sand flies in Jajarm County, North Khorasan Province by Temporal scale, 2017

Species	May	June	July	August	September	October	November	December
Richness (*S*)	4	5	4	7	7	6	7	3
Abundance (*N*)	17	52	62	151	109	69	49	8
Menhinick (*D*_*M*g_)	0.97	0.69	0.50	0.56	0.67	0.72	1	1.06
Margalef (*D*_*Mn*_)	1.05	1.01	0.72	1.19	1.27	1.18	1.54	0.96
Shannon-Weiner (*H′*)	1	0.92	0.95	1.13	1.11	1.22	1.37	1.08
Simpson (*D*)	0.43	0.45	0.42	0.40	0.44	0.36	0.32	0.34
Evenness (*J’*)	0.68	0.50	0.65	0.44	0.43	0.56	0.56	0.98

**Fig. 3:**
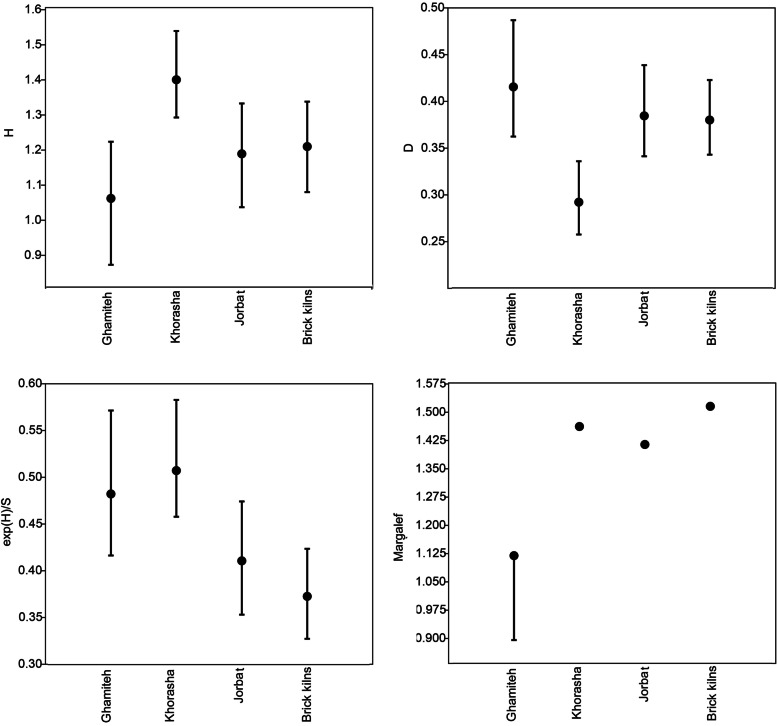
Comparison of Biodiversity Indices [Shannon (*H′*), Dominance (*D*), evenness (exp (*H / S*), Margalef (*D*_*Mn*_)] between Ghamiteh, Khorasha, Jorbat and Brick kilns, 2017

**Fig. 4:**
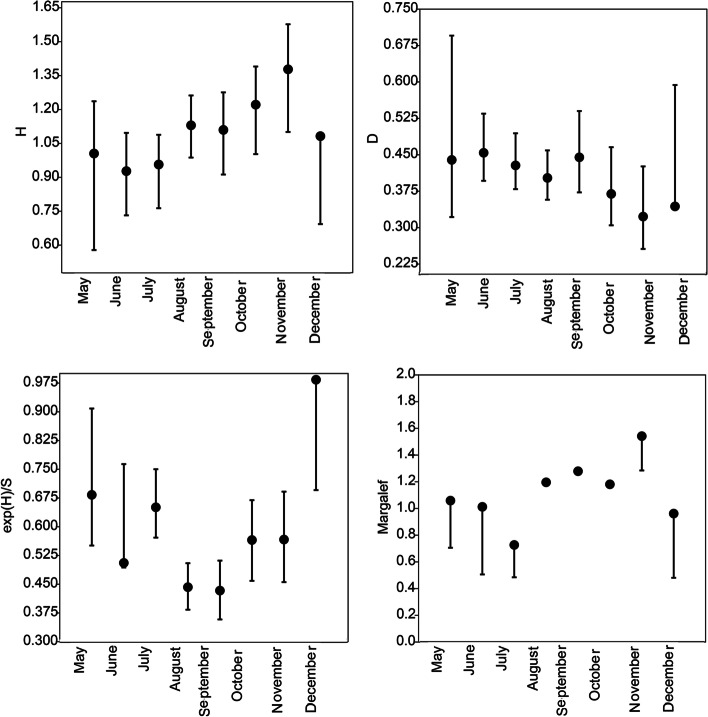
Comparison of Biodiversity Indices [Shannon (*H′*), Dominance (*D*), evenness (exp (*H / S*), Margalef (*D*_*Mn*_)] between May to December, 2017

Comparison of biodiversity indices showed that there is a significant difference between these indices in spatial and temporal scales (*p* < 0.05) (Figs. 3 and 4). The highest species diversity was found in Khorasha and November due to maximal richness, diversity and also relatively high evenness (Tables 5 and 6).

**Fig. 5:**
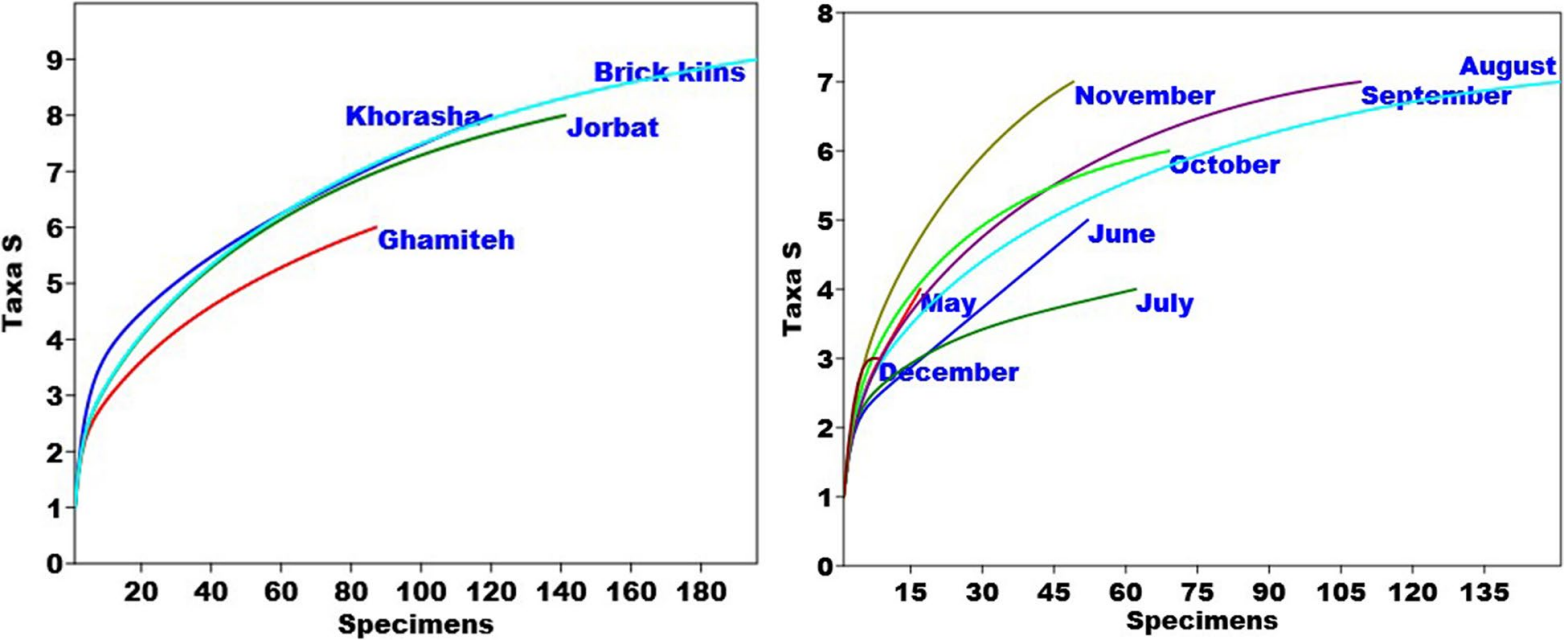
Refraction curve at 95% confidence interval, based on species richness at spatial and temporal scales, 2017

The rarefaction curves show the stability of the number of species in each sample (horizontal axis shows the number of individuals and vertical axis shows the number of expected species yielded from the method), almost all rarefaction curves (at the spatial scale) indicates reaching the asymptotic line. This curve tends to stabilize with nine species in Brick kilns and November. In May, the curve did not reach the asymptotic line. More sampling efforts are likely to be required to increase the richness (Fig. 5).

**Fig. 6:**
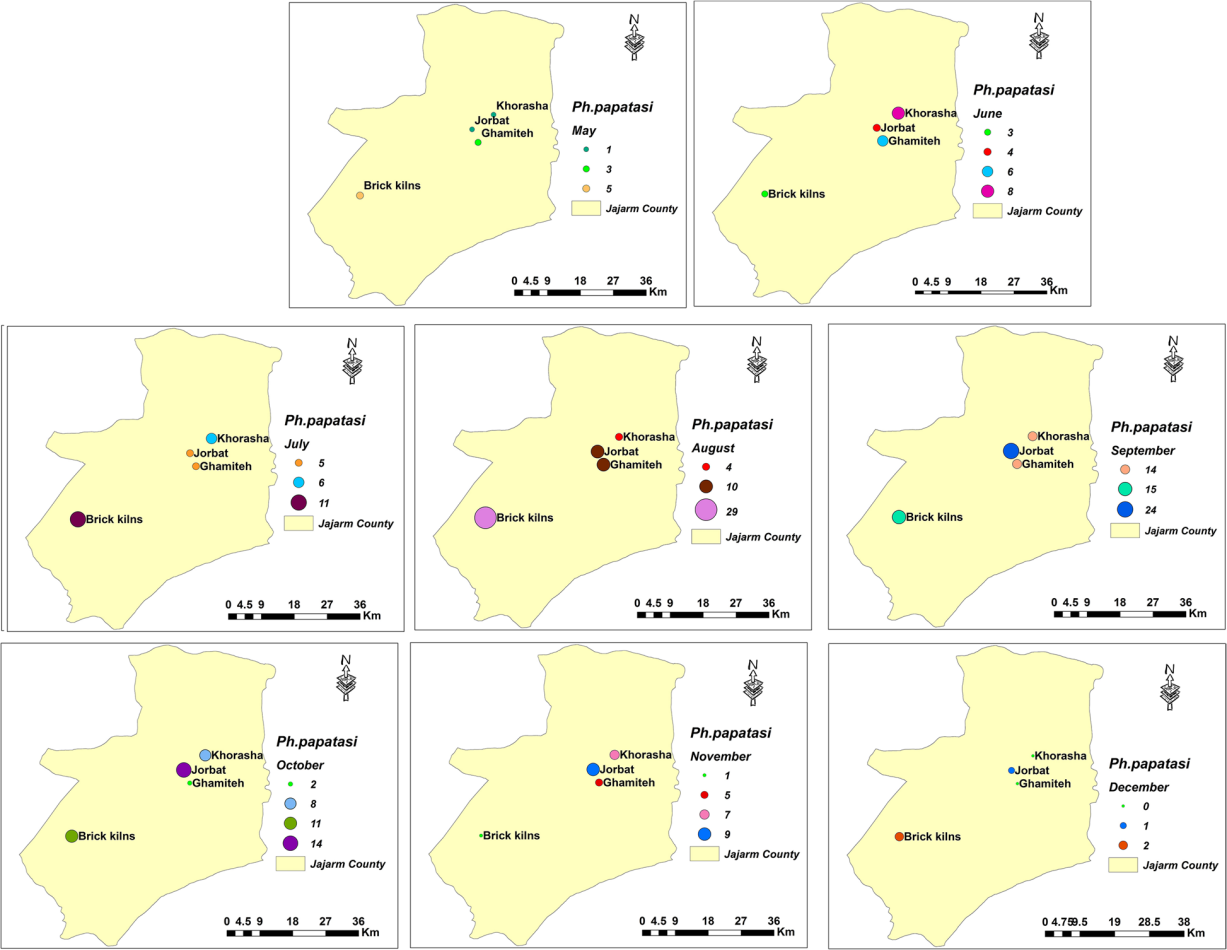
Spatial and temporal distribution of *Ph. papatasi* in Jajarm County, North Khorasan Province, between May to December, 2017

Spatial and temporal distribution of *Ph. papatasi,* as the most abundant specie in the study area, is mapped in Fig. 6. It showed Brick kilns and Jorbat as urban and semi urban areas had more frequency of the main vector of zoonotic cutaneous leishmaniasis in 4 months.

## Discussion

Leishmaniasis is a global health problem due to the significant gap in our understanding of sand fly ecology and unsuccessful control measures [36]. The behavior, biology and ecology of sand flies vary in areas with diverse ecosystems that can prevent control measures. It seems that the best way to reduce the effects on human health is to avoid contact between humans and vectors. Given the high incidence of CL in different cities of North Khorasan Province (average 237.8/100000 people), which is 7 times the annual incidence rate of CL in Iran [25], the establishing reemergence of ZCL in the northeastern provinces of Iran strengthens. Therefore, a basic understanding of the ecological aspects of the native species of the area helps us to properly control the vectors and reduce the burden of disease. The study is considered as the first research on diversity, dominance, seasonal activity, distribution and synanthropic index of sand flies in Jajarm County, southwest of North Khorasan.

In the present study, 5 species of *Phlebotomus* and 3 species of *Sergentomyia* were collected and identified. *Phlebotomus papatasi* and *Se. sintoni* are in the Eudominant class and are collected both indoors and outdoors. These species were mostly trapped from outdoor environments, indicating the exophilic and exophagic behavior in the region. *Phlebotomus papatasi* is associated with the epidemiology of leishmaniasis [25], *Se. sintoni* is believed to prefer feeding on lizards [37]. In accordance with the present study, these species have been introduced as the most abundant species in other studies in Iran [18, 22, 38, 39].

*Phlebotomus papatasi* is known as the main vector of native and non-native foci of ZCL in Iran [18, 40] and neighboring countries [41], incriminated to prefer semi-arid regions [38], which is consistent with the present study and the findings of other researchers who believe that high precipitation is a limiting factor in species distribution [42, 43]. This may be the reason for the abundance of species in the area.

The results indicated that phlebotomine sand flies are mostly present in the warm season, their monthly activity begins in early May and reaches its peak in August and ends in late December in the study area. In contrast, monthly population fluctuations of sand flies were reported to start in late May and finish in late October with two peaks of activity in early July and another in early August by Aghaie-Afshar et al. in Kerman Province [44] and Mawloudi et al. in Paveh County, west of Iran [45]. This difference in the onset of activity may be due to various climatic factors, milder climate can increase the duration of the activity season as mentioned in a study in Bushehr County, southern Iran, where the activity of sand flies started in early April and ended in early January, with their highest activity occurring in early July [46]. However the most prevalent species, *Ph. papatasi* and *Se. sintoni*, in the present study showed a mono-modal trend with a major peak in September and August, respectively, which is in agreement with other researchers’ findings [39]. Other species had lower monthly population densities, which may be due to the specific ecological niches of these species.

There is a significant difference between the sex ratio (Male/female) of sand flies in our research (*p* < 0.005). The sex ratio was up to 0.77 and 0.37 in Yazd Province [39] and was 5.95 and 1.04 in Ilam Province [47] for *Ph. papatasi* and *Se. sintoni* as the dominant species, respectively. In the present study, it was 2.26 and 0.06 for these species, respectively. In general, females were predominated over males. In contrast, in other studies in different parts of Iran, the number of males was always higher than females [48–50]. It seems to be due to differences in sampling methods.

The synanthropic index of sand flies was between + 6.25 and + 38.9 in the study area, which strengthens the tendencies of species to reproduce or grow in the human environment. This index was between − 91.18 and − 69.84 in Khuzestan Province [22]. Barata et al. reported a range of 0.4 to 100 for the synanthropic index of sand flies in Brazil [51]. In general, the index ranges from − 100 to + 100 [52]. *Sergentomyia sintoni* showed higher synanthropic behavior than *ph. papatasi*, possibly due to changes in the ecological needs and behavioral habits of the species, which require further studies in the future. The synanthropic index was not debatable for other species due to low population density.

There was a significant difference in diversity indices in spatial and temporal scales in the study area (Figs. 3 and 4, *p* < 0.05). The highest levels of richness were observed in Brick kilns in August, September and November. Commonly, diversity is positively correlated to species richness [20]. The fact is that although the richness was high, higher diversity was observed in Khorasha and November. This is because the biodiversity index is influenced by two other factors, including species evenness and dominance. The low or high rates of these factors can influence the biodiversity index [53–55]. Since species richness is influenced by sampling intensity, a standard rarefaction curve is used to confirm adequacy in sampling efforts at temporal and spatial scales by reaching the asymptotic line. The curve indicated that further sampling efforts may be needed to assess satisfaction with species richness in May. It may also reflect the fact that some species would start to emerge a little later in spring. There is little data about the biodiversity of Iranian sand flies, indices for other species of insects were studied in northern Iran [54, 55]. Findings of this study showed higher biodiversity (Mean: H ‘=1.21, J’ = 0.29) in the area compared to the northeast (H’ = 0.527–1.033, J’ = 0.345–0.380) (18) and northwest (H’ = 0.4131, J’ = 0.5309) [42] as well as lower in East Azerbaijan Province (H’ = 1.413–1.918) [23], and the Provinces of East and West Azerbaijan and Ardabil (H’ = 1.68–2.30, J’ = 0.73–0.88) [38]. Decreased species diversity in urban and semi-urban areas compared to natural (Khorasha) can also be due to the increase in population of dominant species such as *Ph. papatasi* and *Se. sintoni*, which are well adapted to human environments (Table 4). These findings are consistent with other studies that typically identify urban areas with species richness and diversity less than natural ecosystems [56, 57].

## Conclusion

This study provides useful data on the ecological aspects of sand flies in endemic foci of CL in Jajarm, North Khorasan, and can be helpful in a species-specific control analysis of sand flies to evaluate the risk of *Leishmania* transmission. Occurrence of *Ph. papatasi*, as Eudominant species, has potential implications for human health in our study area [25], highlighting the need for regular health education programs, along with systematic surveillance of sand flies, human and rodent communities.

## Data Availability

The datasets used and/or analyzed during the current study available from the corresponding author on reasonable request.
